# Endoscopic Conduit Harvest - A New Standard in Coronary Artery Bypass Surgery

**DOI:** 10.1186/1749-8090-10-S1-A11

**Published:** 2015-12-16

**Authors:** Maria Cannoletta, Alan Soo, Caroline Revlas, Marimel Olivar, Anthony De Souza, Rashmi Yadav, Richard Trimlett

**Affiliations:** 1 Department of Cardiac Surgery, Royal Brompton Hospital, London, UK

## Background/Introduction

Coronary artery bypass grafting (CABG) remains an index procedure in cardiac surgery. Despite the increasing use of arterial conduits, the great saphenous vein remains the most widely used conduit due to its ease of harvest, availability and versatility. Traditional open harvesting technique (OVH) is associated with significant wound - related morbidity and therefore an endoscopic minimally invasive harvesting technique is increasingly employed. In our institution, endoscopic vein harvest (EVH) is now performed as a routine and here we report our mid-term results.

## Aims/Objectives

We review the results since the inception of this technique.

## Method

EVH was introduced in our institution in November 2009. Data was collected from November 2009 to March 2014. This technique was introduced and performed initially by two consultants and subsequently, following training, by the surgical care practitioners (SCP). Currently, this technique is performed by one consultant and two surgical care practitioners.

## Results

From November 2009 to March 2014, 2334 patients underwent CABG. Initially, in 2010, EVH was employed in 49% of patients. This steadily increases to 54% in 2011 and 73% in 2012. In 2014, 90% of the veins harvested were removed endoscopically.

Patients who underwent EVH have significantly lower rate of leg wound related complications compared to OVH (OVH 3% vs EVH 0.82%, p < 0.0001). There was no statistical difference in 30 days mortality between the two groups (OVH 3.5% vs EVH 2.2%, p = 0.07). Overall survival was 93.1% in OVH and 95.9% in EVH (p = 0.16). There was no difference in reoperation rate (p = 0.69).

## Discussion/Conclusion

The introduction of EVH have no adverse effects on outcome and mortality in the short to medium term and significantly reduced the incidence of leg wound infections compared to traditional open harvesting technique.

